# Tat is a multifunctional viral protein that modulates cellular gene expression and functions

**DOI:** 10.18632/oncotarget.15174

**Published:** 2017-02-07

**Authors:** Evan Clark, Brenda Nava, Massimo Caputi

**Affiliations:** ^1^ Charles E. Schmidt College of Medicine, Florida Atlantic University, Boca Raton, FL, USA

**Keywords:** HIV-1, Tat, transcription, gene regulation

## Abstract

The human immunodeficiency virus type I (HIV-1) has developed several strategies to condition the host environment to promote viral replication and spread. Viral proteins have evolved to perform multiple functions, aiding in the replication of the viral genome and modulating the cellular response to the infection. Tat is a small, versatile, viral protein that controls transcription of the HIV genome, regulates cellular gene expression and generates a permissive environment for viral replication by altering the immune response and facilitating viral spread to multiple tissues. Studies carried out utilizing biochemical, cellular, and genomic approaches show that the expression and activity of hundreds of genes and multiple molecular networks are modulated by Tat via multiple mechanisms.

## INTRODUCTION

Expression of the HIV-1 genome is regulated by a combination of viral and cellular factors. The viral protein Tat (trans-activator of transcription) modulates the activity of the viral promoter. Tat recognizes a short-stem loop structure, known as the transactivation response element (TAR), located at the 5’ terminus of the viral transcript. Tat binding activates the transcription complex that assembles onto the viral promoter, leading to a strong increase in viral transcripts. In addition to promoting viral transcription Tat regulates the expression of cellular genes, modulating key pathways and mechanisms to generate an environment that favors the production and spread of HIV.

Leukocytes regulate the host response to viral infection and are the main cell type targeted by HIV. Tat modulates leukocytes activation, proliferation and activity, thus compromising immune functions and aiding in the progression of the disease. Tat is also released by the infected cells and can be absorbed by bystander cells inducing physiological changes in cells usually not infected such as neurons and endothelial cells. These activities contribute to the development of pathologies associated with the viral infection. Although Tat's functions in viral transcription have been thoroughly studied, less is known on its role as a mediator of physiological processes. This review will focus on the functions of this protein in the regulation of cellular gene expression, modulation of the immune response, and viral pathogenesis.

## TAT STRUCTURAL FEATURES AND FUNCTIONS

Tat is a small basic protein coded by two exons whose length varies between 99 and 103 amino acids with the predominant form being 101 residues. Despite the virus’ high mutation rate, Tat is relatively well conserved in all primate lentiviruses [1]. Tat's primary sequence can be divided into five domains, defined on the basis of amino acid distribution and their conservation within homologous proteins from other lentiviruses: a proline rich acidic N-terminus (aa 1-21), a cysteine-rich region (aa 22-37), a hydrophobic core region (aa 38-48), an arginine-rich basic domain (aa 49- 57), and a C-terminal domain. The first 72 aa are encoded by a first exon while the C-terminus, coded by a second exon, appears to be dispensable for transactivation but retains a role in pathogenesis *in vivo* [2], possibly by modulating expression of MHC class I allowing the virus to persist in the infected host [3], and has a higher variability in viral sequences isolated from patients. The first 57 residues of the protein modulate most of the interactions with its cellular partners; the arginine rich domain (aa 48-57) contains the protein transduction domain (PTD), the nuclear localization signal and the protein's RNA binding domain [4] while the N-terminal 48 amino acids are sufficient for the binding to the cyclin T1 (CycT1) component of the positive transcription elongation factor (P-TEFb) [5].

Structural studies indicate that when free in solution Tat's structure is highly flexible, does not exhibit obvious elements and relies mainly on solvent polarity [6, 7]. Although Tat is an intrinsically disordered protein, this does not prevent its tight binding to the TAR RNA sequence, and cellular partners such as CycT1 [8]. In addition, crystallography studies indicate that the lack of a rigid structure allows the formation of high-affinity complexes with various partners (proteins or lipids), each involving a different specific conformation of Tat [5].

## TAT REGULATION OF HIV-1 TRANSCRIPTION

Transcription regulation of the HIV-1 genome is mediated by RNA polymerase II (RNAPII) and a combination of basal and promoter specific factors [9, 10]. The viral promoter is located within the 5’ long terminal repeat (LTR) of the viral genome and contains two Sp1 binding motifs and two nuclear factor NF-kB binding sites, which serve to regulate the initiation rate of viral transcription [9]. In the absence of Tat, the basal activity of the RNAPII complex assembled onto the viral promoter is extremely low. Furthermore, shortly after transcription initiation, the assembly of two multi-subunit complexes, the negative elongation factor (NELF) and the DRB sensitivity-inducing factor (DSIF), results in RNAPII pausing and the production of abortive short viral transcripts [11, 12]. Tat overcomes this block and increases the rate of transcription by catalyzing the recruitment of the P-TEFb complex, which is composed of cellular CycT1 and the Cyclin-Dependent Kinase 9 (CDK9), and binding to a bulge within the stem loop structure of the TAR RNA, located within the short paused transcript. The binding of Tat to P-TEFb induces conformational changes in CDK9 that constitutively activates the enzyme [5, 13]. Tat extracts and repositions P-TEFb from cellular complexes, such as the Brd4:P-TEFb complex [14], the super elongation complex (SEC) [15, 16] and the 7SK small nuclear ribonucleoproteins (7SK snRNP), which associates with the promoter and functions to sequester P-TEFb in an inactive conformation until the kinase activity of the factor is needed [15].

The activated P-TEFb mediates a complex set of phosphorylation events that modify both positive and negative cellular elongation factors to activate viral transcription by: i) triggering the rearrangement and release of components of the paused transcription-elongation complexes by phosphorylating the NELF-E component of NELF and the STP5 component of DSIF [11, 17] and ii) phosphorylating the C-terminal domain (CTD) of RNAPII to increase the polymerase processivity [18]. Additionally, Tat stimulates the assembly of new transcription complexes by directing the recruitment of TATA box binding protein to the LTR promoter [19] and modulates the phosphorylation of a number of transcription factors including SP1, CREB, the alpha subunit of eukaryotic initiation factor 2 (eIF2a), and NF-kB [20].

Tat's role in viral transcription is not limited to the phosphorylation of promoter associated factors, it also facilitates the recruitment of chromatin-modifying enzymes with histone acetyltransferase (HAT) activity, which induce the acetylation of histones H3 and H4 and relieve the repression exerted on the viral promoter by nucleosomes [21, 22]. In addition, Tat itself is a substrate for acetylation by HATs. Acetylation of lys50 promotes the dissociation of Tat from the TAR sequence in the early phases of elongation and recruitment of the SWI/SNF chromatin-remodeling complex [23], which synergize with the p300 acetyltransferase to remodel the nucleosome and activate transcription [24].

## TAT SECRETION AND INTERNALIZATION

Tat is secreted by the infected cells, accumulates in the extracellular environment and is uptaken by neighboring cells, affecting their gene expression and functions. The mechanism of Tat secretion is poorly defined and appears to depend on a leaderless secretory pathway that is independent from the endoplasmic reticulum and the Golgi apparatus [25] although other mechanisms including direct binding and penetration of the plasma membrane and the exosome biogenesis pathways might be utilized. Tat secretion is highly active allowing its concentration to reach the nanomolar range in infected T cell supernatants in culture, in the sera of HIV-1 infected individuals [26] and in the cerebrospinal fluid (CSF) of virologically controlled individuals undergoing antiretroviral therapy (ART) [27, 28].

Extracellular Tat contributes to HIV-1 pathogenesis by modulating lymphocyte functions, promoting cell migration, and exerting cytopathic effects on leukocytes and neural cells [29]. The presence of Tat in the extracellular environment induces the growth and locomotion of primary endothelial cells in Kaposi's sarcoma (KS) lesions in AIDS patients [30, 31]. Tat competes with the basic fibroblast growth factor (bFGF) for binding to heparan sulfate proteoglycans (HSPGs). The majority of the bFGF is found bound to HSPGs within the cellular matrix and at the cell surface with only a small amount free in solution. Tat increases the availability of soluble bFGF in the KS lesion, which promotes spindle and endothelial cell growth [32, 33] and up-regulates the receptors for fibronectin and vitronectin (integrins α5β1 and αvβ3 respectively), thus increasing the adhesion signals the cells require in order to grow in response to bFGF [34]. In addition, Tat secreted from the infected cells can exert a chemo attractant function for macrophages, monocytes and dendritic cells. Stretches of residues within the cysteine-rich region and the hydrophobic core display similarity with key residues in β-chemokines, which are required for chemokine receptor binding and signal transduction [35, 36].

Although Tat can interact with several factors within the extracellular matrix and on the cellular surface, much of the functions assigned to this protein depend on its ability to penetrate the plasma membrane of uninfected bystander cells. Tat can transduce most cell types, reaching the nucleus to regulate the expression of cellular genes, this activity is dependent on the arginine-rich basic domain of the protein [37, 38]. Tat basic domain, known as the protein transduction domain (PTD), facilitates the trafficking of Tat through the plasma membrane and functions as a cell penetrating peptide when conjugated to a protein cargo [39]. A nuclear localization signal (GRKKR), which mediates translocation of Tat into the cell nucleus, is also found within the PTD. The precise mechanism through which Tat penetrates the plasma membrane appears to be dependent of its ability to recognize multiple binding sites at the cell surface. Endocytic receptors, such as the lipoprotein receptor-related protein (LRP), C-X-C chemokine receptor type 4 (CXCR4) and HSPGs [40–42], allow Tat internalization via two endocytic mechanisms: i) a caveolar pathway and ii) coated pits in an AP-2/clathrin/dynamin 2 dependent pathway [43]. It is still not clear if the clathrin or the caveolar endocytic pathways are both utilized *in-vivo* or if they are dependent on the cell type since T cells do not express caveolin, thus can only utilize the clathrin dependent uptake mechanism.

## REGULATION OF CELLULAR GENES EXPRESSION

Early work showed that cellular processes such as DNA damage repair [44], target cell recruitment [45, 46] and apoptosis [47–49] are modulated by Tat, indicating a direct role for this protein in cellular gene expression. The search for Tat's cellular targets led to the characterization of multiple genes coding for cytokines [50, 51], cell cycle-related proteins [52–54], surface [55–57] and chemokine receptors [58, 59], mRNA processing factors, [60] and enzymes [61]. This lengthy gene list suggests that this viral protein can deeply modify the cellular environment to promote viral replication and disease progression. Although the precise mechanism by which Tat regulates most cellular genes has yet to be characterized, three distinct mechanisms have been defined (Table 1): i) transcription activation by binding to TAR-like sequences in the 5′-untranslated region of nascent RNA; ii) transcription modulation by binding to the promoter region of the target gene and; iii) transcription regulation by interaction with key transcription factors.

**Table 1:** Mechanisms of Tat mediated cellular gene expression

Mechanism	Regulated Genes	Reference
Binding to TAR-like RNA sequence	IL-6 TNF-β	[62, 63] [64,65,66]
Binding to promoter region	MAP2K6 MAP2K3 IRF7 PTEN PPP2R1B PPP2R5E c-Rel	[46] [46] [46] [68, 69] [68, 69] [68, 69] [70]
Interaction with transcription factors	IL2 IL2Rα OGG1 LMP2 CD69 VAV3 ADCYAP1 FAM46C	[71] [71] [44] [72] [73] [73] [73] [73]

Three key mechanisms have been proposed for Tat's modulation of cellular gene expression.

Tumor necrosis factor beta (TNF-β) and Interleukin-6 (IL-6) are examples of genes whose expression is regulated by the binding of Tat onto their 5’ UTR. IL-6 is a pro-inflammatory cytokine, alterations in its expression pattern affect the differentiation of lymphoid cells [62] and are linked to pathologies associated with clinical AIDS that include B cell lymphoma, Kaposi's sarcoma and severe psoriasis [62, 63]. Tat modulates IL-6 transcription by binding a sequence present in the stem-loop structure of the IL-6 leader RNA. Immunoprecipitation and yeast two-hybrid assays confirmed that Tat interacts with the C/EBP transcription factors and increases their assembly onto the IL-6 promoter [62, 63]. A similar mechanism has also been observed in the TNF-β gene [64]. Computational and mutational analyses of the TNF-β promoter indicate strong similarities with the viral LTR. Additionally, a TAR-like structure, present within the 5’ UTR of the primary transcript, is required for Tat-dependent TNF-β expression [65, 66].

Genome based experimental approaches suggest that the expression of a large number of genes is regulated by Tat assembling onto their promoter region. Kim and colleagues [46] utilized chromatin immunoprecipitation assays to identify 308 gene promoters that are bound by Tat in immature dendritic cells and in monocyte-derived macrophages. Tat-dependent expression was experimentally confirmed for three of these promoters, MAP2K6, MAP2K3, and IRF7. Tat-dependent up-regulation of MAP2K6, MAP2K3, and IRF7 induced activation of several interferon stimulated genes (ISG) via the Janus kinase-STAT (Jak-STAT) and the mitogen-activated protein (Kinase) (MAPK) pathways [67]. A similar ChIP-on-Chip and RNA expression analysis approach in Jurkat cells showed that Tat binds and regulates the promoters of three genes required in the mechanism regulating CD4+ T cell apoptosis, the phosphatase and tensin homolog (PTEN), and two of the subunits of the protein phosphatase 2A (PP2A), PPP2R1B and PPP2R5E [46, 68, 69]. Tat binding to a promoter sequence can also negatively affect gene expression as shown in the c-Rel promoter where Tat can occupy the NF-kB binding site inhibiting the binding of NF-kB causing the down-regulation of c-Rel expression [70].

Finally, Tat has been shown to modulate gene expression by interfering with the activity of known transcription regulators. Tat can inhibit the activity of the special AT-rich sequence binding protein 1 (SATB1), which recruits histone deacetylase 1 (HDAC1) to repress transcription from the IL-2 and IL-2Rα promoters. Studies carried out in a mouse model indicate that Tat binding to SATB1 displaces HDAC1 inducing de-repression of these promoters [71] and expression of IL-2 and its receptor. Similarly, binding of the negative transcription factor AP-4 to the 8-oxoguanine-DNA glycosylase-1 (OGG1) promoter is inhibited by AP-4 interaction with Tat, resulting in an increase in the enzyme expression [44]. Since OGG1 is responsible for the repair of oxidatively damaged DNA, Tat appears to play a role in the maintenance of the genetic integrity of proviral and host cell DNAs. Furthermore, Tat can repress the transcription of low-molecular-mass polypeptide 2 (LMP2) by inhibiting the complex formed at the promoter between the positive transcription factor STAT1 and the interferon regulatory factor 1 (IRF1) [72]. More recently, a genome wide approach carried out in primary T cells and model cell lines has revealed that over 400 genes might be regulated by the interaction of Tat with the transcription factors ETS1, a T cell master regulator, RUNX1 and GATA3 [73]. In this study, genes up-regulated by Tat appear to play a positive role in T cell activation and promote viral replication and spread while genes down-regulated by Tat may function in weakening the immune response.

## REGULATION OF miRNA EXPRESSION

In addition to directly modulating the transcription of cellular genes Tat plays a role in post-transcriptional gene regulation through the alteration of the expression and activity of small non-coding RNAs. Preliminary studies showed that Tat inhibits miRNA function by binding to the Dicer protein in an RNA-dependent manner [74, 75]. Recent work carried out utilizing miRNA profiling showed that Tat can tightly bind at least 18 miRNA and downregulate 10 of them [76]. Most of these miRNAs are likely involved in key neural processes, such as axonal guidance and glucocorticoid signaling, thus confirming a role for Tat in HIV neuropathogenesis. In addition Tat deregulates neuronal functions by reducing the level of miR-196a, which causes an increase in the Abelson murine leukemia (c-Abl) protein, which phosphorylates the pro-apoptotic transcription factor p73, thus regulating its activity [77].

**Figure 1:**
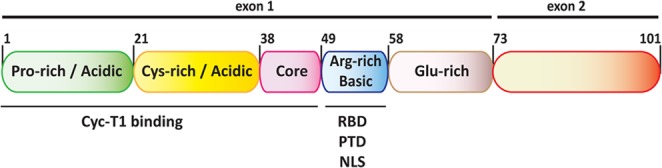
Diagram of the functional domains of HIV-1 Tat Tat is subdivided into six functional domains. The first 5 domains, coded by the first exon, are sufficient for trans-activation of viral transcription and modulate most of the interactions with Tat cellular partners. The arginine rich basic domain functions as a RNA binding domain (RBD), a protein transduction domain (PTD) and nuclear localization signal (NLS). The second exon codes for the C-terminal domain that, which contains a tripeptide RGD motif, does not appear to be required for Tat functions in cell culture but might contribute to viral pathogenesis *in-vivo*.

Tat can also induce the expression of miR-132, which targets a set of genes required for proper neural growth (MeCP2, BDNF, p250GAP, BDNF). miR-132 induction is associated with the increase phosphorylation and activity of the cAMP response element-binding (CREB) protein [78]. Less understood is the mechanism by which Tat upregulates the expression of miR-101, which results in the downregulation of VE-cadherin, a master regulator of brain endothelial permeability [79].

## MODULATION OF THE IMMUNE SYSTEM

HIV primarily infects cells that express the CD4 receptor: T cells, monocytes/macrophages, and dendritic cells are the main viral targets. Infection of CD4+ cells leads to chronic immune activation and dysfunctional cytokine production. This promotes apoptosis, alters the response to pathogens and results in the weakening of the immune system, facilitating viral replication, persistence and progression to clinical AIDS.

CD4+ T cells are classified in three subsets: i) naïve T cells that have not yet been exposed to an antigen, ii) helper T cells that have been activated by a specific antigen and produce cytokines that can be toxic to the target cells or can stimulate other T and B cells and iii) memory T cells, which have encountered an antigen during a prior infection and at a second exposure can mount a faster and stronger immune response.

Naïve T cells are quiescent, non-dividing, and can be activated upon interaction with an antigen-presenting cell (APC) through the simultaneous engagement of the T cell receptor (TCR) and a co-stimulatory molecule, like CD28, by the major histocompatibility complex present on the antigen presenting cells [80]. Tat can mediate T cell activation through an alternative pathway, which utilizes VEGFR2 and is independent from the engagement of the TCR. Microarray assays performed on primary T cells have characterized a panel of 94 genes that are deregulated when Tat is present in the culture media. Among these genes IL-8 and IL-1β activate VEGFR2 gene expression inducing T cell activation [28]. Since the virus preferentially infects activated T cells Tat might help promote viral propagation, when present in the extracellular environment, by penetrating uninfected naïve T cells and modulating the activity of other bystander cells. In addition, Tat can up-regulate the expression of the surface CXC-chemokine receptor 4 (CXCR4), a co-receptor required for viral entry, in resting CD4+ T cells, and increase their susceptibility to HIV infection [59].

A different subset of T cells, that expresses the CD8 receptor, is able to recognize and lyse infected cells [81]. Both T cell subtypes, CD4+ and CD8+, mature from pluripotent stem cells via a complex selection process that yields different subpopulations that express either CD4 or CD8 receptors in combination with TCRs [82]. Tat blocks the maturation of CD4+ and CD8+ T cells by deregulating the expression of cytokines, required in the T cell maturation process, in a Tat-producing transgenic mouse model [83]. Tat can also affect the activity of mature CD8+ T cells by stimulating the down-regulation of the IL-7 receptor [84], a heterodimeric receptor complex composed of CD127 and CD132, which stimulates CD8+ T cell proliferation and potentiates their cytolytic activity [85]. Tat is internalized by uninfected CD8+ cells and promotes the removal and degradation of CD127. This reduces the IL-7 receptor activity and impairs cell proliferation and functions, contributing to a reduced cell mediated immunity.

**Figure 2:**
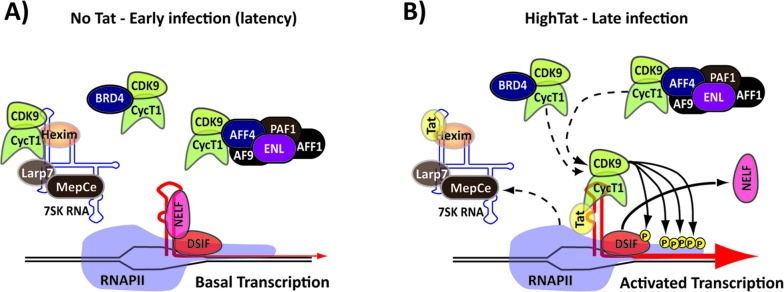
Tat activates viral transcription **A**. In the early stage of viral infection, in the absence of Tat, transcription is limited by a pausing complex (NELF, DSIF) that assembles onto the nascent transcript. P-TEFb is sequestered in multiple complexes. **B**. During the later stages of the viral infection Tat is present at high concentrations and induces relocation of P-TEFb from diverse nucleoplasmic complexes to the nascent transcript within the paused RNAPII complex by its binding to TAR. This triggers activation of the P-TEFb kinase and hyper-phosphorylation of the RNAPII CTD and the NELF/DSIF complex.

The production and functions of cytokines are also affected by Tat [86, 87]. The expression of TNF-α, IL-6 and IL-8, three pro-inflammatory cytokines, and IL-10, an immunosuppressive cytokine, are increased by Tat binding to the complex formed by the toll-like receptor 4 (TLR4) and its cofactor MD2. Binding of Tat to TLR4-MD2 activates a series of signaling cascades, which include MAP kinases, PKC, and NF-kB, that lead to the production of TNF-α and IL-10 [88–90]. Furthermore, studies carried out in human astrocytes and microglial cells showed that Tat expression leads to a dramatic increase in the secretion of several chemokines (CCL2, CCL3, CCL4, CCL5, CXCL8, CXCL10 and XCL1) [91], although the molecular basis of this regulatory mechanism is not yet understood.

## REGULATION OF APOPTOSIS

Apoptosis is a highly regulated and controlled process of programmed cell death, which is activated in response to internal and external stimuli. The apoptotic process, together with cytopathic effects caused by the virus and the immune system, are the main causes of the decline in the T cell population in infected patients. A number of signals can trigger the initiation of the enzyme cascade that regulates this process. Tat can interfere with the apoptotic process by: i) targeting the mitochondrial pathway, which compromises the integrity of the mitochondrial membrane, and ii) targeting the direct signal transduction pathway, which transduces the apoptotic signal via the TNF-induced or Fas-Fas ligand-mediated path [68, 92].

**Figure 3:**
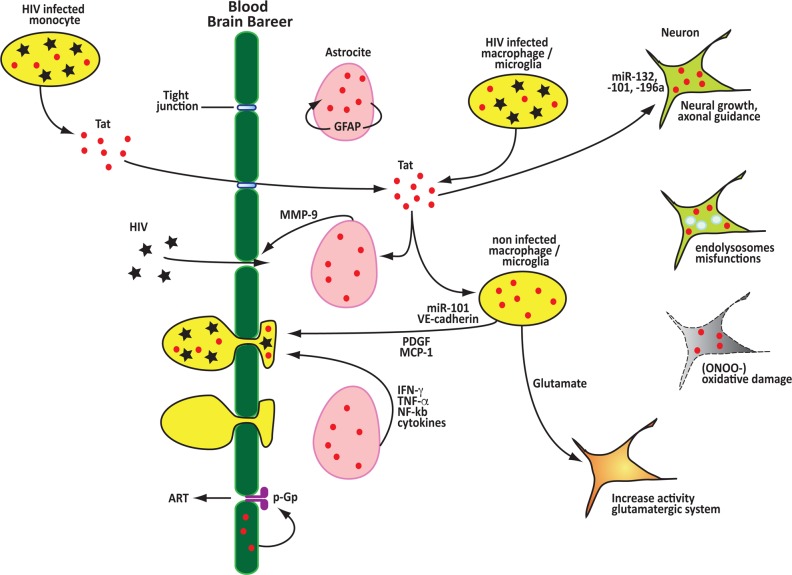
Tat contributes to the disruption of the blood brain barrier, neuroinflammation and neurotoxicity Extracellular Tat can passively cross the BBB decreasing the expression of tight junctions proteins and inducing the expression of factors that promote the disruption of the BBB. After crossing the BBB Tat is efficiently taken up by multiple cells, including neurons and astrocytes, this results in the release of several soluble factors that promote neuroinflammation and function as monocyte chemoattractant facilitating the invasion of the CNS by infected monocytes and macrophages. Tat directly induces neuronal toxicity by modulating the expression of miRNAs, disrupting endolysosomes functions, promoting oxidative damage and inpairing the functions of the glutamatergic neurotransmission system. Tat can also promote the remaval of anti retro viral drugs from the CNS by upregulating the P-glycoprotein (P-gp) pump.

Tat can interact with the αβ-tubulin dimer, polymerized microtubules [93], and LIS1, a scaffolding protein that assists in regulating microtubule formation dynamics [94], leading to the activation of the mitochondria-dependent pathway. Tat can also directly up-regulate the expression of PTEN and PP2A [68], which act to decrease the phosphorylation of Forkhead Box O3 (FOXO3a), a factor that promotes T cell apoptosis when in its unphosphorylated state. Tat has also been shown to modulate the Fas-Fas ligand-mediated pathway by increasing the expression of Fas ligand (FasL) [48], which induces apoptosis upon binding to the Fas receptor [95–97] and indoleamine 2,3-dioxygenase (IDO), an enzyme that catalyzes the degradation L-tryptophan and contributes to IFN-γ induced apoptosis, triggering both the mitochondrial and the direct signal pathways [98–100].

Differently from apoptosis, autophagy is a cellular process that allows for the degradation and recycling of cellular components in response to specific stress signals utilizing a double-membraned vesicle known as an autophagosome, which fuses with a lysosome to degrade its content. Although some line of evidence indicate that Tat might induce autophagy by enhancing the level of the pro-autophagy factor BAG3 [101] Tat can also negatively regulate autophagy by decreasing the phosphorylation of the transcription factor STAT1, which correlates with a decrease in the expression of microtubule-associated protein 1 light chain 3 beta (LC3B), a key component of the autophagosome [102], thus causing an overall decrease in autophagy.

## TAT ROLE IN NEUROCOGNITIVE DISORDERS

Although the virus primarily targets the cells of the immune system HIV can also penetrate and infect the components of the central nervous system (CNS). HIV gains access to the CNS mainly through infected monocytes and macrophages that cross the blood brain bareer (BBB) and subsequently infects microglial cells and astrocytes [29], causing a variety of neurological disorders that include HIV-associated dementia (HAD), minor neurocognitive disorder (MND), and asymptomatic neurocognitive impairment (ANI). These pathological conditions have now been collectively grouped and termed as HIV-associated neurocognitive disorder (HAND) [103]. These disorders are caused by damage in the neural tissue due to both viral cytopathic effects and the inflammatory response to the infection [104].

Tat has been shown to be present in relatively high level in the CNS of patients receiving ART [27, 28] and plays a critical role in the breach of the BBB. Tat can passively cross the BBB [105], decreasing the expression of tight junction proteins, such as Claudin-5 [106], adherens junction proteins, such as VE-cadherin [79] and membrane extracellular matrix proteins [107], while promoting the expression of inflammatory molecules and of the matrix metalloproteinase 9 (MMP-9), as shown in both *in-vitro* and mice models [108–110]. The disruption of the BBB is one of the main causes of neuroinflammation, which results in an influx of inflammatory cells into the CNS. After crossing the BBB extracellular Tat is efficiently taken up by multiple cells, including neurons, which results in the release of several soluble factors that promote neuroinflammation. Tat up-regulates expression of the platelet-derived growth factor (PDGF), a potent inducer of the chemokine monocyte chemoattractant protein 1 (MCP-1) [111]. Induction of MCP-1 triggers an influx of HIV-infected monocytes, which leads to an abnormal increase in the number of astrocytes due to the destruction of nearby neurons and subsequent inflammatory responses [112]. Chemokines can also contribute to neuroinflammation and loss of neurons [113, 114]. Studies carried out in human astrocytes revealed that the combined activities of Tat and the pro-inflammatory cytokines IFN-γ and TNF-α resulted in the generation of reactive oxygen species (ROS), activation of Jnk, Erk1/2, and Akt pathways, and subsequent activation of NF-kB, leading to the increase in expression of CXCL10 which contributes to neuroinflammation [115, 116]. Tat has also been shown to up-regulate expression of the glial fibrillary acidic protein (GFAP) [117], which is associated with astrocyte proliferation, dysfunction and reduced neuronal plasticity.

Tat has been also shown to promote neuronal toxicity and synaptodendritic injury. Mouse models expressing HIV-1 Tat have shown histological changes similar to those observed in HAND patients [118, 119]. Tat causes changes in the structure and function of endolysosomes, which play an early role in HIV-1 induced neuronal damage [120]. Tat promotes an increase in endolysosomes size, disrupts their membrane integrity, elevates their PH, thus decreasing their activity, and inhibit autophagy. Furthermore, when present in the extracellular environment Tat can penetrate neural cells and increases levels of reactive oxygen species and lipid peroxidation by activating the inducible nitric oxide synthase (iNOS) to produce nitric oxide (NO), which is converted in the highly reactive peroxynitrite (ONOO-) [121]. The increase in reactive oxygen species within neural cells results in extensive damage and promotes neurodegenerative disease.

Tat neurotoxicity is also connected to its effects on the glutamatergic neurotransmission system. An excess in Glutamate, an excitatory neurotransmitter essential for learning, memory, and synaptic plasticity, leads to neuron death. Tat increases microglial glutamate release, elevating extracellular glutamate levels [122], while impairing glutamate reuptake by astrocytes [117] and further promoting glutamate functions by binding and phosphorylating the glutamate *N*-methyl-D-aspartate (NMDA) receptor [123, 124].

An additional role of Tat in the development of neurocognitive disorders might be connected to the low levels of ART observed in the brain of patients [125]. Tat has been shown to increase the promoter activity and expression of the P-glycoprotein (P-gp) [126], a pump present in the cells forming the BBB and required for the transport of small molecule out of the brain. It is plausible that Tat might help in establishing a brain reservoir of HIV by up-regulating P-gp, thus removing ART from the CNS.

## CONCLUSIONS

HIV-1 infection hijacks the cellular machinery and regulates cellular signaling pathways to promote its replication and modulate the host immune response. Understanding in detail how the virus and its proteins affect the cells on a molecular level will greatly help in designing proper therapeutic strategies that will allow us to alleviate pathologies and secondary complications associated with the current antiretroviral therapy. Tat can interact with the host cell genome, cellular messengers, transcription factors and several proteins to alter expression of cellular genes and generate a permissive environment for viral replication. Recent studies have also suggested that Tat may play a role in the establishment and control of a viral reservoir, which is mainly constituted by infected resting and memory CD4+ T cells [127]. Although the Tat level in infected resting T cells is very low it clearly plays an important role for expression of HIV in resting cells as mutations in Tat result in lower levels of HIV expression [128].

Given Tat's ability to translocate through cellular membranes and transduce uninfected cells, it will be informative to determine how this protein modulates and reprograms gene expression in multiple cell types and tissues to understand the global changes it causes. Furthermore, the precise mechanisms by which Tat interacts and regulates the activity of master transcription regulators such as ETS1 and other cellular proteins is still unclear, more studies are needed to define such mechanisms and explore novel therapeutic approaches, as suggested by the development of Tat-based vaccines [129]. Ultimately, the full understanding of the complex biological pathways regulated by this multi-tasking protein can be applied to fields different from HIV-1 research, thus improving our understanding of complex gene regulatory networks.

## References

[1] Viglianti GA, Mullins JI (1988). Functional comparison of transactivation by simian immunodeficiency virus from rhesus macaques and human immunodeficiency virus type 1. Journal of virology.

[2] Verhoef K, Bauer M, Meyerhans A, Berkhout B (1998). On the role of the second coding exon of the HIV-1 Tat protein in virus replication and MHC class I downregulation. AIDS research and human retroviruses.

[3] Howcroft TK, Strebel K, Martin MA, Singer DS (1993). Repression of MHC class I gene promoter activity by two-exon Tat of HIV. Science (New York, N.Y.).

[4] Puglisi JD, Chen L, Blanchard S, Frankel AD (1995). Solution structure of a bovine immunodeficiency virus Tat-TAR peptide-RNA complex. Science New York, N.Y..

[5] Tahirov TH, Babayeva ND, Varzavand K, Cooper JJ, Sedore SC, Price DH (2010). Crystal structure of HIV-1 Tat complexed with human P-TEFb. Nature.

[6] Bayer P, Kraft M, Ejchart A, Westendorp M, Frank R, Rosch P (1995). Structural studies of HIV-1 Tat protein. Journal of molecular biology.

[7] Shojania S, O'Neil JD (2006). HIV-1 Tat is a natively unfolded protein: the solution conformation and dynamics of reduced HIV-1 Tat-(1-72) by NMR spectroscopy. The Journal of biological chemistry.

[8] Zhang J, Tamilarasu N, Hwang S, Garber ME, Huq I, Jones KA, Rana TM (2000). HIV-1 TAR RNA enhances the interaction between Tat and cyclin T1. The Journal of biological chemistry.

[9] Pereira LA, Bentley K, Peeters A, Churchill MJ, Deacon NJ (2000). A compilation of cellular transcription factor interactions with the HIV-1 LTR promoter. Nucleic acids research.

[10] Brady J, Kashanchi F (2005). Tat gets the “green” light on transcription initiation. Retrovirology.

[11] Wada T, Takagi T, Yamaguchi Y, Ferdous A, Imai T, Hirose S, Sugimoto S, Yano K, Hartzog GA, Winston F, Buratowski S, Handa H (1998). DSIF, a novel transcription elongation factor that regulates RNA polymerase II processivity, is composed of human Spt4 and Spt5 homologs. Genes & development.

[12] Wada T, Orphanides G, Hasegawa J, Kim DK, Shima D, Yamaguchi Y, Fukuda A, Hisatake K, Oh S, Reinberg D, Handa H (2000). FACT relieves DSIF/NELF-mediated inhibition of transcriptional elongation and reveals functional differences between P-TEFb and TFIIH. Molecular cell.

[13] Isel C, Karn J (1999). Direct evidence that HIV-1 Tat stimulates RNA polymerase II carboxyl-terminal domain hyperphosphorylation during transcriptional elongation. Journal of molecular biology.

[14] Yang Z, Yik JH, Chen R, He N, Jang MK, Ozato K, Zhou Q (2005). Recruitment of P-TEFb for stimulation of transcriptional elongation by the bromodomain protein Brd4. Molecular cell.

[15] Sobhian B, Laguette N, Yatim A, Nakamura M, Levy Y, Kiernan R, Benkirane M (2010). HIV-1 Tat assembles a multifunctional transcription elongation complex and stably associates with the 7SK snRNP. Molecular cell.

[16] He N, Liu M, Hsu J, Xue Y, Chou S, Burlingame A, Krogan NJ, Alber T, Zhou Q (2010). HIV-1 Tat and host AFF4 recruit two transcription elongation factors into a bifunctional complex for coordinated activation of HIV-1 transcription. Molecular cell.

[17] Fujinaga K, Irwin D, Huang Y, Taube R, Kurosu T, Peterlin BM (2004). Dynamics of human immunodeficiency virus transcription: P-TEFb phosphorylates RD and dissociates negative effectors from the transactivation response element. Molecular and cellular biology.

[18] Parada CA, Roeder RG (1996). Enhanced processivity of RNA polymerase II triggered by Tat-induced phosphorylation of its carboxy-terminal domain. Nature.

[19] Raha T, Cheng SW, Green MR (2005). HIV-1 Tat stimulates transcription complex assembly through recruitment of TBP in the absence of TAFs. PLoS biology.

[20] Romani B, Engelbrecht S, Glashoff RH (2010). Functions of Tat: the versatile protein of human immunodeficiency virus type 1. The Journal of general virology.

[21] Marcello A, Zoppe M, Giacca M (2001). Multiple modes of transcriptional regulation by the HIV-1 Tat transactivator. IUBMB life.

[22] Easley R, Van Duyne R, Coley W, Guendel I, Dadgar S, Kehn-Hall K, Kashanchi F (2010). Chromatin dynamics associated with HIV-1 Tat-activated transcription. Biochimica et biophysica acta.

[23] Treand C, du Chene I, Bres V, Kiernan R, Benarous R, Benkirane M, Emiliani S (2006). Requirement for SWI/SNF chromatin-remodeling complex in Tat-mediated activation of the HIV-1 promoter. The EMBO journal.

[24] Mahmoudi T, Parra M, Vries RG, Kauder SE, Verrijzer CP, Ott M, Verdin E (2006). The SWI/SNF chromatin-remodeling complex is a cofactor for Tat transactivation of the HIV promoter. The Journal of biological chemistry.

[25] Nickel W, Rabouille C (2009). Mechanisms of regulated unconventional protein secretion. Nat Rev Mol Cell Biol.

[26] Barillari G, Ensoli B (2002). Angiogenic effects of extracellular human immunodeficiency virus type 1 Tat protein and its role in the pathogenesis of AIDS-associated Kaposi's sarcoma. Clin Microbiol Rev.

[27] Mediouni S, Darque A, Baillat G, Ravaux I, Dhiver C, Tissot-Dupont H, Mokhtari M, Moreau H, Tamalet C, Brunet C, Paul P, Dignat-George F, Stein A (2012). Antiretroviral therapy does not block the secretion of the human immunodeficiency virus tat protein. Infect Disord Drug Targets.

[28] Johnson TP, Patel K, Johnson KR, Maric D, Calabresi PA, Hasbun R, Nath A (2013). Induction of IL-17 and nonclassical T-cell activation by HIV-Tat protein. Proceedings of the National Academy of Sciences of the United States of America.

[29] Bagashev A, Sawaya BE (2013). Roles and functions of HIV-1 Tat protein in the CNS: an overview. Virol J.

[30] Ensoli B, Barillari G, Salahuddin SZ, Gallo RC, Wong-Staal F (1990). Tat protein of HIV-1 stimulates growth of cells derived from Kaposi's sarcoma lesions of AIDS patients. Nature.

[31] Ensoli B, Buonaguro L, Barillari G, Fiorelli V, Gendelman R, Morgan RA, Wingfield P, Gallo RC (1993). Release, uptake, and effects of extracellular human immunodeficiency virus type 1 Tat protein on cell growth and viral transactivation. Journal of virology.

[32] Barillari G, Sgadari C, Palladino C, Gendelman R, Caputo A, Morris CB, Nair BC, Markham P, Nel A, Sturzl M, Ensoli B (1999). Inflammatory cytokines synergize with the HIV-1 Tat protein to promote angiogenesis and Kaposi's sarcoma via induction of basic fibroblast growth factor and the alpha v beta 3 integrin. J Immunol.

[33] Barillari G, Sgadari C, Fiorelli V, Samaniego F, Colombini S, Manzari V, Modesti A, Nair BC, Cafaro A, Sturzl M, Ensoli B (1999). The Tat protein of human immunodeficiency virus type-1 promotes vascular cell growth and locomotion by engaging the alpha5beta1 and alphavbeta3 integrins and by mobilizing sequestered basic fibroblast growth factor. Blood.

[34] Benelli R, Barbero A, Ferrini S, Scapini P, Cassatella M, Bussolino F, Tacchetti C, Noonan DM, Albini A (2000). Human immunodeficiency virus transactivator protein (Tat) stimulates chemotaxis, calcium mobilization, and activation of human polymorphonuclear leukocytes: implications for Tat-mediated pathogenesis. J Infect Dis.

[35] Campbell GR, Watkins JD, Singh KK, Loret EP, Spector SA (2007). Human immunodeficiency virus type 1 subtype C Tat fails to induce intracellular calcium flux and induces reduced tumor necrosis factor production from monocytes. Journal of virology.

[36] Albini A, Benelli R, Giunciuglio D, Cai T, Mariani G, Ferrini S, Noonan DM (1998). Identification of a novel domain of HIV tat involved in monocyte chemotaxis. The Journal of biological chemistry.

[37] Jeang KT, Xiao H, Rich EA (1999). Multifaceted activities of the HIV-1 transactivator of transcription, Tat. The Journal of biological chemistry.

[38] Mann DA, Frankel AD (1991). Endocytosis and targeting of exogenous HIV-1 Tat protein. The EMBO journal.

[39] Schwarze SR, Hruska KA, Dowdy SF (2000). Protein transduction: unrestricted delivery into all cells?. Trends in cell biology.

[40] Liu HX, Chew SL, Cartegni L, Zhang MQ, Krainer AR (2000). Exonic splicing enhancer motif recognized by human SC35 under splicing conditions. Molecular and cellular biology.

[41] Tyagi M, Rusnati M, Presta M, Giacca M (2001). Internalization of HIV-1 tat requires cell surface heparan sulfate proteoglycans. The Journal of biological chemistry.

[42] Signoret N, Oldridge J, Pelchen-Matthews A, Klasse PJ, Tran T, Brass LF, Rosenkilde MM, Schwartz TW, Holmes W, Dallas W, Luther MA, Wells TN, Hoxie JA, Marsh M (1997). Phorbol esters and SDF-1 induce rapid endocytosis and down modulation of the chemokine receptor CXCR4. The Journal of cell biology.

[43] Debaisieux S, Rayne F, Yezid H, Beaumelle B (2012). The ins and outs of HIV-1 Tat. Traffic (Copenhagen, Denmark).

[44] Imai K, Nakata K, Kawai K, Hamano T, Mei N, Kasai H, Okamoto T (2005). Induction of OGG1 gene expression by HIV-1 Tat. The Journal of biological chemistry.

[45] Izmailova E, Bertley FM, Huang Q, Makori N, Miller CJ, Young RA, Aldovini A (2003). HIV-1 Tat reprograms immature dendritic cells to express chemoattractants for activated T cells and macrophages. Nature medicine.

[46] Kim N, Kukkonen S, Martinez-Viedma Mdel P, Gupta S, Aldovini A (2013). Tat engagement of p38 MAP kinase and IRF7 pathways leads to activation of interferon-stimulated genes in antigen-presenting cells. Blood.

[47] Selliah N, Finkel TH (2001). Biochemical mechanisms of HIV induced T cell apoptosis. Cell death and differentiation.

[48] Campbell GR, Pasquier E, Watkins J, Bourgarel-Rey V, Peyrot V, Esquieu D, Barbier P, de Mareuil J, Braguer D, Kaleebu P, Yirrell DL, Loret EP (2004). The glutamine-rich region of the HIV-1 Tat protein is involved in T-cell apoptosis. The Journal of biological chemistry.

[49] Giacca M (2005). HIV-1 Tat, apoptosis and the mitochondria: a tubulin link?. Retrovirology.

[50] Buonaguro L, Barillari G, Chang HK, Bohan CA, Kao V, Morgan R, Gallo RC, Ensoli B (1992). Effects of the human immunodeficiency virus type 1 Tat protein on the expression of inflammatory cytokines. Journal of virology.

[51] Ott M, Lovett JL, Mueller L, Verdin E (1998). Superinduction of IL-8 in T cells by HIV-1 Tat protein is mediated through NF-kappaB factors. J Immunol.

[52] Li CJ, Wang C, Friedman DJ, Pardee AB (1995). Reciprocal modulations between p53 and Tat of human immunodeficiency virus type 1. Proceedings of the National Academy of Sciences of the United States of America.

[53] Longo F, Marchetti MA, Castagnoli L, Battaglia PA, Gigliani F (1995). A novel approach to protein-protein interaction: complex formation between the p53 tumor suppressor and the HIV Tat proteins. Biochemical and biophysical research communications.

[54] Roy S, Katze MG, Parkin NT, Edery I, Hovanessian AG, Sonenberg N (1990). Control of the interferon-induced 68-kilodalton protein kinase by the HIV-1 tat gene product. Science (New York, N.Y.).

[55] Caldwell RL, Egan BS, Shepherd VL (2000). HIV-1 Tat represses transcription from the mannose receptor promoter. J Immunol.

[56] Willard-Gallo KE, Furtado M, Burny A, Wolinsky SM (2001). Down-modulation of TCR/CD3 surface complexes after HIV-1 infection is associated with differential expression of the viral regulatory genes. European journal of immunology.

[57] Fogel S, Guittaut M, Legrand A, Monsigny M, Hebert E (1999). The tat protein of HIV-1 induces galectin-3 expression. Glycobiology.

[58] Huang L, Bosch I, Hofmann W, Sodroski J, Pardee AB (1998). Tat protein induces human immunodeficiency virus type 1 (HIV-1) coreceptors and promotes infection with both macrophage-tropic and T-lymphotropic HIV-1 strains. Journal of virology.

[59] Secchiero P, Zella D, Capitani S, Gallo RC, Zauli G (1999). Extracellular HIV-1 tat protein up-regulates the expression of surface CXC-chemokine receptor 4 in resting CD4+ T cells. J Immunol.

[60] Calzado MA, Sancho R, Munoz E (2004). Human immunodeficiency virus type 1 Tat increases the expression of cleavage and polyadenylation specificity factor 73-kilodalton subunit modulating cellular and viral expression. Journal of virology.

[61] Gavioli R, Gallerani E, Fortini C, Fabris M, Bottoni A, Canella A, Bonaccorsi A, Marastoni M, Micheletti F, Cafaro A, Rimessi P, Caputo A, Ensoli B (2004). HIV-1 tat protein modulates the generation of cytotoxic T cell epitopes by modifying proteasome composition and enzymatic activity. J Immunol.

[62] Ambrosino C, Ruocco MR, Chen X, Mallardo M, Baudi F, Trematerra S, Quinto I, Venuta S, Scala G (1997). HIV-1 Tat induces the expression of the interleukin-6 (IL6) gene by binding to the IL6 leader RNA and by interacting with CAAT enhancer-binding protein beta (NF-IL6) transcription factors. The Journal of biological chemistry.

[63] Scala G, Ruocco MR, Ambrosino C, Mallardo M, Giordano V, Baldassarre F, Dragonetti E, Quinto I, Venuta S (1994). The expression of the interleukin 6 gene is induced by the human immunodeficiency virus 1 TAT protein. J Exp Med.

[64] Sastry KJ, Reddy HR, Pandita R, Totpal K, Aggarwal BB (1990). HIV-1 tat gene induces tumor necrosis factor-beta (lymphotoxin) in a human B-lymphoblastoid cell line. The Journal of biological chemistry.

[65] Buonaguro L, Buonaguro FM, Giraldo G, Ensoli B (1994). The human immunodeficiency virus type 1 Tat protein transactivates tumor necrosis factor beta gene expression through a TAR-like structure. Journal of virology.

[66] Brother MB, Chang HK, Lisziewicz J, Su D, Murty LC, Ensoli B (1996). Block of Tat-mediated transactivation of tumor necrosis factor beta gene expression by polymeric-TAR decoys. Virology.

[67] Kukkonen S, Martinez-Viedma Mdel P, Kim N, Manrique M, Aldovini A (2014). HIV-1 Tat second exon limits the extent of Tat-mediated modulation of interferon-stimulated genes in antigen presenting cells. Retrovirology.

[68] Kim N, Kukkonen S, Gupta S, Aldovini A (2010). Association of Tat with promoters of PTEN and PP2A subunits is key to transcriptional activation of apoptotic pathways in HIV-infected CD4+ T cells. PLoS pathogens.

[69] Marban C, Su T, Ferrari R, Li B, Vatakis D, Pellegrini M, Zack JA, Rohr O, Kurdistani SK (2011). Genome-wide binding map of the HIV-1 Tat protein to the human genome. PloS one.

[70] Dhamija N, Choudhary D, Ladha JS, Pillai B, Mitra D (2015). Tat predominantly associates with host promoter elements in HIV-1-infected T-cells - regulatory basis of transcriptional repression of c-Rel. The FEBS journal.

[71] Kumar PP, Purbey PK, Ravi DS, Mitra D, Galande S (2005). Displacement of SATB1-bound histone deacetylase 1 corepressor by the human immunodeficiency virus type 1 transactivator induces expression of interleukin-2 and its receptor in T cells. Molecular and cellular biology.

[72] Remoli AL, Marsili G, Perrotti E, Gallerani E, Ilari R, Nappi F, Cafaro A, Ensoli B, Gavioli R, Battistini A (2006). Intracellular HIV-1 Tat protein represses constitutive LMP2 transcription increasing proteasome activity by interfering with the binding of IRF-1 to STAT1. The Biochemical journal.

[73] Reeder JE, Kwak YT, McNamara RP, Forst CV, D'Orso I (2015). HIV Tat controls RNA Polymerase II and the epigenetic landscape to transcriptionally reprogram target immune cells. Elife.

[74] Bennasser Y, Jeang KT (2006). HIV-1 Tat interaction with Dicer: requirement for RNA. Retrovirology.

[75] Bennasser Y, Le SY, Benkirane M, Jeang KT (2005). Evidence that HIV-1 encodes an siRNA and a suppressor of RNA silencing. Immunity.

[76] Sardo L, Vakil PR, Elbezanti W, El-Sayed A, Klase Z (2016). The inhibition of microRNAs by HIV-1 Tat suppresses beta catenin activity in astrocytes. Retrovirology.

[77] Bagashev A, Mukerjee R, Santerre M, Del Carpio-Cano FE, Shrestha J, Wang Y, He JJ, Sawaya BE (2014). Involvement of miR-196a in HIV-associated neurocognitive disorders. Apoptosis.

[78] Rahimian P, He JJ (2016). HIV-1 Tat-shortened neurite outgrowth through regulation of microRNA-132 and its target gene expression. J Neuroinflammation.

[79] Mishra R, Singh SK (2013). HIV-1 Tat C modulates expression of miRNA-101 to suppress VE-cadherin in human brain microvascular endothelial cells. J Neurosci.

[80] Tseng SY, Dustin ML (2002). T-cell activation: a multidimensional signaling network. Current opinion in cell biology.

[81] Gulzar N, Copeland KF (2004). CD8+ T-cells: function and response to HIV infection. Current HIV research.

[82] Germain RN (2002). T-cell development and the CD4-CD8 lineage decision. Nat Rev Immunol.

[83] Fiume G, Scialdone A, Albano F, Rossi A, Tuccillo FM, Rea D, Palmieri C, Caiazzo E, Cicala C, Bellevicine C, Falcone C, Vecchio E, Pisano A (2015). Impairment of T cell development and acute inflammatory response in HIV-1 Tat transgenic mice. Sci Rep.

[84] Faller EM, Sugden SM, McVey MJ, Kakal JA, MacPherson PA (2010). Soluble HIV Tat protein removes the IL-7 receptor alpha-chain from the surface of resting CD8 T cells and targets it for degradation. J Immunol.

[85] Faller EM, McVey MJ, MacPherson PA (2014). IL-7 receptor recovery on CD8 T-cells isolated from HIV+ patients is inhibited by the HIV Tat protein. PloS one.

[86] Rappaport J, Joseph J, Croul S, Alexander G, Del Valle L, Amini S, Khalili K (1999). Molecular pathway involved in HIV-1-induced CNS pathology: role of viral regulatory protein, Tat. Journal of leukocyte biology.

[87] Reinhart TA (2003). Chemokine induction by HIV-1: recruitment to the cause. Trends in immunology.

[88] Ben Haij N, Leghmari K, Planes R, Thieblemont N, Bahraoui E (2013). HIV-1 Tat protein binds to TLR4-MD2 and signals to induce TNF-alpha and IL-10. Retrovirology.

[89] Gee K, Angel JB, Mishra S, Blahoianu MA, Kumar A (2007). IL-10 regulation by HIV-Tat in primary human monocytic cells: involvement of calmodulin/calmodulin-dependent protein kinase-activated p38 MAPK and Sp-1 and CREB-1 transcription factors. J Immunol.

[90] Ben Haij N, Planes R, Leghmari K, Serrero M, Delobel P, Izopet J, BenMohamed L, Bahraoui E (2015). HIV-1 Tat Protein Induces Production of Proinflammatory Cytokines by Human Dendritic Cells and Monocytes/Macrophages through Engagement of TLR4-MD2-CD14 Complex and Activation of NF-kappaB Pathway. PloS one.

[91] Kim BO, Liu Y, Zhou BY, He JJ (2004). Induction of C chemokine XCL1 (lymphotactin/single C motif-1 alpha/activation-induced, T cell-derived and chemokine-related cytokine) expression by HIV-1 Tat protein. J Immunol.

[92] Zheng L, Yang Y, Guocai L, Pauza CD, Salvato MS (2007). HIV Tat protein increases Bcl-2 expression in monocytes which inhibits monocyte apoptosis induced by tumor necrosis factor-alpha-related apoptosis-induced ligand. Intervirology.

[93] Epie N, Ammosova T, Sapir T, Voloshin Y, Lane WS, Turner W, Reiner O, Nekhai S (2005). HIV-1 Tat interacts with LIS1 protein. Retrovirology.

[94] Chen D, Wang M, Zhou S, Zhou Q (2002). HIV-1 Tat targets microtubules to induce apoptosis, a process promoted by the pro-apoptotic Bcl-2 relative Bim. The EMBO journal.

[95] Westendorp MO, Frank R, Ochsenbauer C, Stricker K, Dhein J, Walczak H, Debatin KM, Krammer PH (1995). Sensitization of T cells to CD95-mediated apoptosis by HIV-1 Tat and gp120. Nature.

[96] Dockrell DH, Badley AD, Villacian JS, Heppelmann CJ, Algeciras A, Ziesmer S, Yagita H, Lynch DH, Roche PC, Leibson PJ, Paya CV (1998). The expression of Fas Ligand by macrophages and its upregulation by human immunodeficiency virus infection. J Clin Invest.

[97] Cohen SS, Li C, Ding L, Cao Y, Pardee AB, Shevach EM, Cohen DI (1999). Pronounced acute immunosuppression *in vivo* mediated by HIV Tat challenge. Proceedings of the National Academy of Sciences of the United States of America.

[98] Planes R, Bahraoui E (2013). HIV-1 Tat protein induces the production of IDO in human monocyte derived-dendritic cells through a direct mechanism: effect on T cells proliferation. PloS one.

[99] Fu X, Lawson MA, Kelley KW, Dantzer R (2011). HIV-1 Tat activates indoleamine 2,3 dioxygenase in murine organotypic hippocampal slice cultures in a p38 mitogen-activated protein kinase-dependent manner. J Neuroinflammation.

[100] Barton C, Davies D, Balkwill F, Burke F (2005). Involvement of both intrinsic and extrinsic pathways in IFN-gamma-induced apoptosis that are enhanced with cisplatin. Eur J Cancer.

[101] Bruno AP, De Simone FI, Iorio V, De Marco M, Khalili K, Sariyer IK, Capunzo M, Nori SL, Rosati A (2014). HIV-1 Tat protein induces glial cell autophagy through enhancement of BAG3 protein levels. Cell cycle (Georgetown, Tex).

[102] Li JC, Au KY, Fang JW, Yim HC, Chow KH, Ho PL, Lau AS (2011). HIV-1 trans-activator protein dysregulates IFN-gamma signaling and contributes to the suppression of autophagy induction. AIDS (London, England).

[103] Mocchetti I, Bachis A, Avdoshina V (2012). Neurotoxicity of human immunodeficiency virus-1: viral proteins and axonal transport. Neurotox Res.

[104] Marcondes MC, Burudi EM, Huitron-Resendiz S, Sanchez-Alavez M, Watry D, Zandonatti M, Henriksen SJ, Fox HS (2001). Highly activated CD8(+) T cells in the brain correlate with early central nervous system dysfunction in simian immunodeficiency virus infection. J Immunol.

[105] Banks WA, Robinson SM, Nath A (2005). Permeability of the blood-brain barrier to HIV-1 Tat. Experimental neurology.

[106] Andras IE, Toborek M (2011). HIV-1-induced alterations of claudin-5 expression at the blood-brain barrier level. Methods in molecular biology (Clifton, N.J.).

[107] Zhong Y, Smart EJ, Weksler B, Couraud PO, Hennig B, Toborek M (2008). Caveolin-1 regulates human immunodeficiency virus-1 Tat-induced alterations of tight junction protein expression via modulation of the Ras signaling. J Neurosci.

[108] Ju SM, Song HY, Lee JA, Lee SJ, Choi SY, Park J (2009). Extracellular HIV-1 Tat up-regulates expression of matrix metalloproteinase-9 via a MAPK-NF-kappaB dependent pathway in human astrocytes. Exp Mol Med.

[109] Xu R, Feng X, Xie X, Zhang J, Wu D, Xu L (2012). HIV-1 Tat protein increases the permeability of brain endothelial cells by both inhibiting occludin expression and cleaving occludin via matrix metalloproteinase-9. Brain Res.

[110] Huang W, Chen L, Zhang B, Park M, Toborek M (2014). PPAR agonist-mediated protection against HIV Tat-induced cerebrovascular toxicity is enhanced in MMP-9-deficient mice. J Cereb Blood Flow Metab.

[111] Bethel-Brown C, Yao H, Hu G, Buch S (2012). Platelet-derived growth factor (PDGF)-BB-mediated induction of monocyte chemoattractant protein 1 in human astrocytes: implications for HIV-associated neuroinflammation. J Neuroinflammation.

[112] Bethel-Brown C, Yao H, Callen S, Lee YH, Dash PK, Kumar A, Buch S (2011). HIV-1 Tat-mediated induction of platelet-derived growth factor in astrocytes: role of early growth response gene 1. J Immunol.

[113] Dhillon NK, Peng F, Ransohoff RM, Buch S (2007). PDGF synergistically enhances IFN-gamma-induced expression of CXCL10 in blood-derived macrophages: implications for HIV dementia. J Immunol.

[114] Asensio VC, Campbell IL (2001). Chemokines and viral diseases of the central nervous system. Adv Virus Res.

[115] Williams R, Dhillon NK, Hegde ST, Yao H, Peng F, Callen S, Chebloune Y, Davis RL, Buch SJ (2009). Proinflammatory cytokines and HIV-1 synergistically enhance CXCL10 expression in human astrocytes. Glia.

[116] Williams R, Yao H, Peng F, Yang Y, Bethel-Brown C, Buch S (2010). Cooperative induction of CXCL10 involves NADPH oxidase: Implications for HIV dementia. Glia.

[117] Zhou BY, Liu Y, Kim B, Xiao Y, He JJ (2004). Astrocyte activation and dysfunction and neuron death by HIV-1 Tat expression in astrocytes. Molecular and cellular neurosciences.

[118] Bruce-Keller AJ, Chauhan A, Dimayuga FO, Gee J, Keller JN, Nath A (2003). Synaptic transport of human immunodeficiency virus-Tat protein causes neurotoxicity and gliosis in rat brain. J Neurosci.

[119] Kim BO, Liu Y, Ruan Y, Xu ZC, Schantz L, He JJ (2003). Neuropathologies in transgenic mice expressing human immunodeficiency virus type 1 Tat protein under the regulation of the astrocyte-specific glial fibrillary acidic protein promoter and doxycycline. Am J Pathol.

[120] Hui L, Chen X, Haughey NJ, Geiger JD (2012). Role of endolysosomes in HIV-1 Tat-induced neurotoxicity. ASN Neuro.

[121] Kim SH, Smith AJ, Tan J, Shytle RD, Giunta B (2015). MSM ameliorates HIV-1 Tat induced neuronal oxidative stress via rebalance of the glutathione cycle. Am J Transl Res.

[122] Gupta S, Knight AG, Gupta S, Knapp PE, Hauser KF, Keller JN, Bruce-Keller AJ (2010). HIV-Tat elicits microglial glutamate release: role of NAPDH oxidase and the cystine-glutamate antiporter. Neurosci Lett.

[123] Haughey NJ, Nath A, Mattson MP, Slevin JT, Geiger JD (2001). HIV-1 Tat through phosphorylation of NMDA receptors potentiates glutamate excitotoxicity. Journal of neurochemistry.

[124] Song L, Nath A, Geiger JD, Moore A, Hochman S (2003). Human immunodeficiency virus type 1 Tat protein directly activates neuronal N-methyl-D-aspartate receptors at an allosteric zinc-sensitive site. J Neurovirol.

[125] Gimenez F, Fernandez C, Mabondzo A (2004). Transport of HIV protease inhibitors through the blood-brain barrier and interactions with the efflux proteins, P-glycoprotein and multidrug resistance proteins. J Acquir Immune Defic Syndr.

[126] Zhong Y, Hennig B, Toborek M (2010). Intact lipid rafts regulate HIV-1 Tat protein-induced activation of the Rho signaling and upregulation of P-glycoprotein in brain endothelial cells. J Cereb Blood Flow Metab.

[127] Razooky BS, Pai A, Aull K, Rouzine IM, Weinberger LS (2015). A hardwired HIV latency program. Cell.

[128] DeMaster LK, Liu X, VanBelzen DJ, Trinite B, Zheng L, Agosto LM, Migueles SA, Connors M, Sambucetti L, Levy DN, Pasternak AO, O'Doherty U (2016). A Subset of CD4/CD8 Double-Negative T Cells Expresses HIV Proteins in Patients on Antiretroviral Therapy. Journal of virology.

[129] Caputo A, Gavioli R, Bellino S, Longo O, Tripiciano A, Francavilla V, Sgadari C, Paniccia G, Titti F, Cafaro A, Ferrantelli F, Monini P, Ensoli F, Ensoli B (2009). HIV-1 Tat-based vaccines: an overview and perspectives in the field of HIV/AIDS vaccine development. International reviews of immunology.

